# Optical Tweezers with Integrated Multiplane Microscopy (OpTIMuM): a new tool for 3D microrheology

**DOI:** 10.1038/s41598-021-85013-y

**Published:** 2021-03-10

**Authors:** Andrew B. Matheson, Lynn Paterson, Amanda J. Wright, Tania Mendonca, Manlio Tassieri, Paul A. Dalgarno

**Affiliations:** 1 Institute of Biological Chemistry, Biophysics and Bioengineering, School of Engineering and Physical Sciences, Heriot Watt University, Edinburgh, EH14 4AS UK; 2 Optics and Photonics Research Group, Faculty of Engineering, University of Nottingham, Nottingham, NG7 2RD UK; 3 Division of Biomedical Engineering, James Watt School of Engineering, University of Glasgow, 76 Oakfield Avenue, Glasgow, G12 8LT UK

**Keywords:** Biological techniques, Biophysics, Materials science, Optics and photonics, Physics

## Abstract

We introduce a novel 3D microrheology system that combines for the first time Optical Tweezers with Integrated Multiplane Microscopy (OpTIMuM). The system allows the 3D tracking of an optically trapped bead, with ~ 20 nm accuracy along the optical axis. This is achieved without the need for a high precision z-stage, separate calibration sample, nor a priori knowledge of either the bead size or the optical properties of the suspending medium. Instead, we have developed a simple yet effective in situ spatial calibration method using image sharpness and exploiting the fact we image at multiple planes simultaneously. These features make OpTIMuM an ideal system for microrheology measurements, and we corroborate the effectiveness of this novel microrheology tool by measuring the viscosity of water in three dimensions, simultaneously.

## Introduction

Microrheology is the study of the flow of matter at micron length scales. It can be performed either in situ of environments commonly inaccessible to conventional bulk rheology techniques (e.g. in vivo^1^), or in vitro requiring only a few microliters of sample volume. This is attractive for biophysical and biomedical studies, where rare or precious samples are often investigated. A common aim of microrheology techniques is to determine the rheological properties of fluids via a statistical mechanics analysis of the trajectory of tracer particles suspended in the sample^2^. These have been successfully used to gather new insights on how living systems such as cells^3,4^, bacteria^5^ and phytoplankton^6^ are affected by the rheological properties of the local environment and vice versa. Microrheology techniques can be broadly classified into two families, ‘passive’ and ‘active’ microrheology depending on whether the motion of the tracer particles is thermally driven or governed by an external force, respectively.

In the specific case of passive microrheology with optical tweezers (MOT), also defined as *hybrid* microrheology because of the presence of a confining force^7^, analysis of the Brownian motion (or less commonly the rotation^8^) of a micron-sized tracer bead reveals (i) the strength of the constraining optical trap and (ii) the viscoelastic properties of the suspending medium directly surrounding the bead^9^. An advantage of MOT when working with fluid samples is the confining force which prevents the tracer bead diffusing out of the field of view, allowing the bead to be tracked for longer periods of time and hence over a wider range of frequencies. MOT has the additional benefit of allowing the positioning of the probe particle at a location of interest within the liquid sample; e.g., close to a boundary wall or next to a cell^6^. The accuracy to which MOT can evaluate the strength of the optical trap and the viscoelastic properties of the suspending medium is directly related to the precision to which the bead position can be detected. This is commonly achieved very effectively in two-dimensions (i.e. in the imaging plane of the microscope, which is commonly equivalent to the x–y plane perpendicular to the laser beam axis) using either a high speed camera or a quadrant photodiode (QPD)^10^. However, optically trapped particles actually experience Brownian motion in 3D, where the 3^rd^ dimension of the motion (coincident with the optical axis, i.e. the z-direction) is largely ignored in microrheology studies, despite its potential importance when characterising non-uniform samples or performing measurements in proximity to a surface parallel to the x–y plane. The different options for 3D imaging of trapped beads are discussed in much detail in the thorough review by Liang et al.^11^. The most commonly used methods for performing 3D imaging are optical sectioning techniques, such as confocal microscopy, where a piezo-electric scanner is used to move either the objective or the sample in the z-direction allowing a 3D image stack to be generated.

These methods are not typically suitable for MOT because they come with the following caveats: (i) when the z-scan is achieved by moving the microscope objective or specialised optics placed directly behind the objective^12,13^ the position of the trapping beam waist and therefore of the optically trapped particle is also altered; (ii) when it is achieved by moving the sample holder, disruptive vibrations may overshadow the thermal fluctuations; and (iii) piezo scanners are commonly limited to speeds ≤ 100 Hz ^14^, which would significantly hinder the highest accessible experimental frequency to which the viscoelastic properties of the material could be deduced. An alternative approach that doesn’t suffer from any of these problems is to use a QPD. The Gouy phase shift obtained from a QPD can allow beads to be effectively tracked in 3D over small distances in z^15,16^. This has been combined with optical trapping to allow for 3D imaging of soft structures, to great effect^17^. However, QPD’s can be challenging to calibrate, and only operate over a short range in z. In the literature, several other imaging techniques have been reported that also extend the tracking of a bead to 3D and, in principle, they could be employed for MOT; these include using electrically tunable lenses^18^, interferometry^19^, and stereo-microscopy^20,21^. One other option is to use holographic microscopy, as demonstrated in the microrheology system of Cheong et al.^22^. This system gives excellent microrheology results, but in doing so replaces the standard illumination with a HeNe laser to produce the holographic image. This makes the system less convenient for probing ultra-local properties in biological systems, where it may be useful to simultaneously use transmission images to see how close the probe bead is to e.g. cells^6^.

The system we present here is based upon multiplane microscopy. Multiplane microscopy is a potent imaging technique that allows us to capture images at multiple different focal planes simultaneously. Conventionally, multiplane microscopy techniques can be classified into two categories: (i) those that split the image with beam splitters and then use lenses to change the focal depth for each beam path^23^, and (ii) those where diffractive optical elements are employed^24^. In this work we have used the latter approach to simultaneously image a single optically trapped microsphere at nine different focal planes and thus perform microrheology measurements in three dimensions. A significant advantage that OpTIMuM has over many of the aforementioned 3D imaging techniques is its simplicity, as it requires only the addition of passive optical components into the optical path between the microscope and the camera. Previous multiplane microscopy work by this group used a look-up table approach to track sub-diffraction limit sized beads in 3D^25^. This is a procedure that, in common with other look-up table approaches requires the generation of a calibration data set on a bead and sample system which must match the optical and the physical properties of the bead being used for measurements. These properties include, bead size, bead refractive index, fluid homogeneity and illumination. Generally the measurements will be carried out using the same tracer bead that the calibration data set was made with.

The ability to gain spatial information in the z-direction makes OpTIMuM a valuable tool for a variety of studies throughout the applied sciences. These include, but are not limited to, exploring the properties of 3D cell cultures^3,26^, elucidating the mechanical proprieties of materials with highly anisotropic structures^27^, and investigating the complex interactions at the solid–liquid interface of planes and/or biochemically treated surfaces^28,29^.

Our approach to 3D localisation may equally be applied to studies of complex interactions and processes involving cells, macromolecules and pathogens^30–33^, where the combination of high frame rates, no moving parts and compatibility with optical trapping would make it highly effective.

## Methods

**Figure 1:**
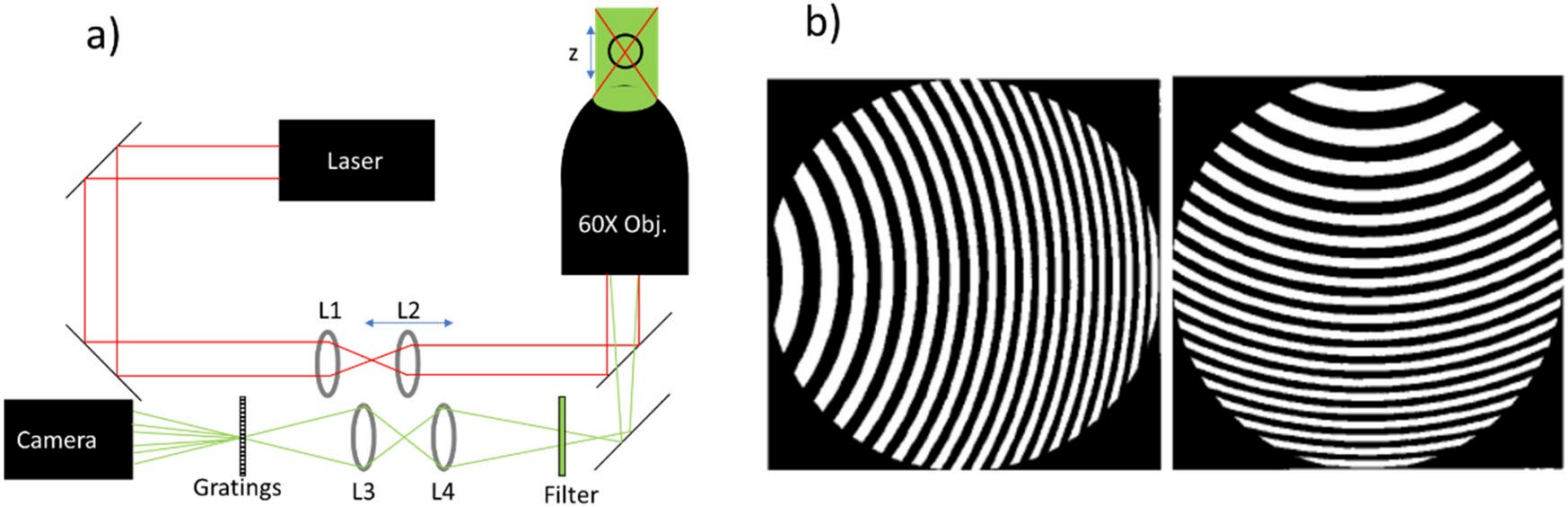
(**a**) Schematic diagram of our optical system. A 60 × objective collects transmitted light (green) and delivers the trap laser (red). Lenses L1 and L2 are used as a beam expander to over-fill the back aperture of the 60X objective. L2 is mounted on a translation stage to allow the position of the focus and hence the trapped particle to be adjusted in z. L3 and L4 are used to create a 4f system with the camera at 4f and grating at 3f from the image plane of the microscope. (**b**) Schematic representation of the etch patterns of the gratings used to split images to perform multiplane microscopy. Two orthogonal gratings with etched surfaces facing each other and placed as close together as possible are necessary to view nine different focal planes.

The system was designed using an Olympus IX73 inverted wide field microscope with an Olympus 60 × water immersion lens (Olympus UPLSAPO60XW) having a numerical aperture (NA) of 1.2. Images were taken in transmission, using a green LED (Thorlabs LED-C13) for the illumination and then filtering the images using a narrow bandpass filter (Thorlabs, FWHM = 3 nm) centred on 532 nm. The narrow filter is required to minimize chromatic aberrations due to the gratings. Images were captured using a Hamumatsu Orca Flash 4.0 camera at 67 frames per second. To test the capability of the z-localisation we used a piezo microscope stage (Mad. City Labs Nanodrive 85) to move the stage in z. We worked in transmission rather than fluorescence to avoid photobleaching the optically trapped particle, which would have had increased the complexity of the z-localisation process. A schematic diagram for the system is shown in Fig. 1a. For the optical trap, we used the output of a 1064 nm laser (Opus, Laser Quantum). In our system, as in most optical tweezer platforms, a beam expander consisting of two lenses (L1 (30 mm) and L2 (200 mm)) ensured that the trapping laser over-fills the back aperture of the objective and makes best use of its NA. By adjusting the position of lens 2 relative to lens 1 via a manually operated Thorlabs CT1translation mount, any bead trapped by the laser would move in the z-direction relative to the planes of the imaging system. Similar configurations have been employed in optical tweezers experiments previously^29^. To avoid the effects of laser heating on the sample we used a 1064 nm laser which is only very weakly absorbed by water and kept trap power ≤ 20 mW throughout.

**Figure 2:**
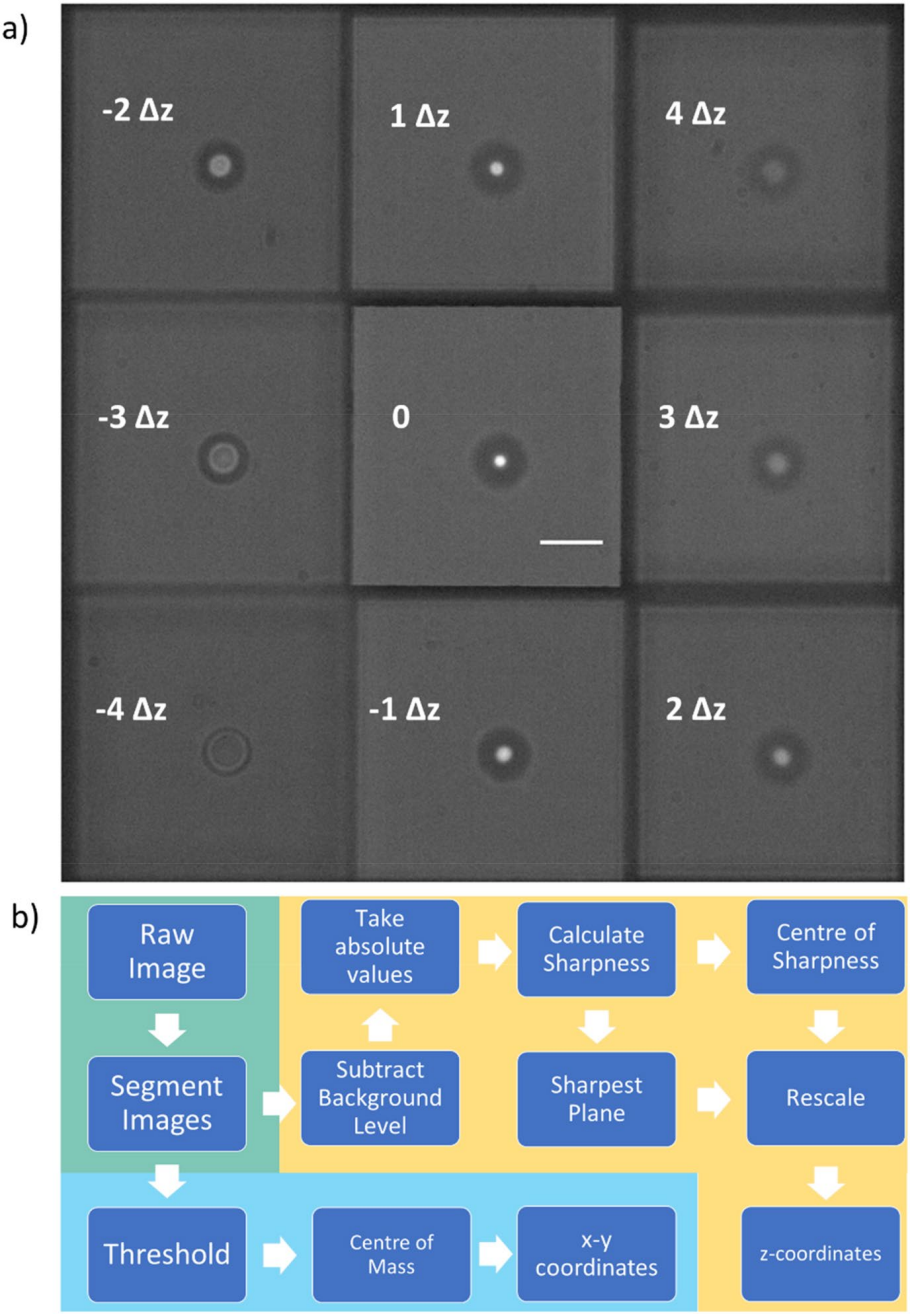
(**a**) A single frame showing a bead of ~ 4 µm radius trapped in gel and imaged at nine depths simultaneously. The top-left corner of each image reports the relative distance between that specific image plane and the plane in the centre (0), in multiples of the plane spacing Δz = 0.88 µm. The scale bar, shown in the central plane (0) is 10 µm. Contrast and brightness have been adjusted for clarity (Matlab 2019b; MathWorks, Natick, MA, https://uk.mathworks.com/products/matlab.html). (**b**) A flowchart of the image processing algorithm used to extract x, y, and z coordinates of the bead. Steps on the blue background relate to x and y localisation, whereas those on the gold background relate to z localisation; those on the green background represent initial processing steps common to x, y and z.

The multiplane system used in this work is similar to the one described in refs.^25,34,35^. To briefly summarise, we have attached a 4f image relay system (consisting of two 300 mm lenses) to the camera output port. The 4f system allows us to place the multiplane grating in the telecentric position and thus maintain a consistent level of magnification in each of the imaging focal planes. The multiplane grating itself is a quadratically distorted diffraction grating etched into a quartz substrate by Photronics UK Ltd. A schematic representation of the etch pattern is shown in Fig. 1b, although the real gratings have a much shorter etch period than shown. The optics underpinning the operation of the multiplane system are described at length in ref^36^, but in simplest terms the object planes from different depths in z are spatially separated on a single camera. A single grating may be used to separate an image into three sub-images, corresponding to the m = 0, ± 1 diffraction orders. By using two gratings with orthogonal etch patterns, it is possible to split a single image into nine different sub-images, which due to the quadritic distortion of the gratings have each corresponding to a different image depth, as shown in Fig. 2a. In our system we have used a relay and grating combination that gives plane separation of Δz = 0.88 µm, a short explanation of why this spacing was chosen can be found in the “Z-localisation” section where we discuss how sharpness may be used to localize the bead in z. The plane spacing was measured by moving fixed beads of varying sizes in different samples and under differing illumination conditions up and down a known distance using the piezo z-stage, and checking the stage-position which maximized the value of Sharpness in each image plane. This characterisation of the plane spacings is performed upon installation of the system, and there is no further need for a high precision stage during measurements, unless the objective or gratings are changed.

The image processing is represented schematically by the flowchart shown in Fig. 2b. To summarise, raw images captured by the camera are segmented into the nine different sub-images each corresponding to a z_plane_ = n Δz, for n =  ± 4,3,2,1,0, where the central plane is n = 0. Each segmented image stack is then processed with the “multithresh” function in Matlab [Matlab 2019b; MathWorks, Natick, MA]. This uses Otsu’s method to find a threshold value which maximises inter-class intensity variance and minimises intra-class intensity variance^37^. This threshold is then applied to the segmented images to create a black background, and the x, y coordinates calculated by performing centre of mass analysis. The segmented images without the threshold are retained, a background level is subtracted, and the absolute value of the pixel intensities taken. These are then used to calculate the z-position of the particle as described in the “Results” section.

The underlying principles of the microrheology analysis performed on the particle trajectory is extensively described in references^9^ and^10^. Briefly, we calculate the normalized mean squared displacement (NMSD) of the bead in x, y and z directions, which is related to the normalized position autocorrelation function (NPAF) by the following equation: $NMSD=1-NPAF=1-\mathrm{exp}\left(-\frac{{\kappa }_{i}\tau }{6\pi \eta r}\right)$, where $\kappa =$ trap stiffness, defined as *κ*_*x*_ = *k*_*B*_*T/* < *x*^*2*^ > (where *x* may be replaced by *y* or *z*, *T* is the absolute temperature and *k*_*B*_ is Boltzmann constant) and $i=x,y,z$. Bead radius is given by *r*, $\tau$ is lag-time and *η* is the unknown fluid viscosity that could be measured as described in the “Results” section. Note that the last equality of the above equation is valid only in the case of Newtonian fluids.

To test our z-localisation method, we used a bead embedded in a stiff agarose gel such that its thermal motion in z is negligible compared to the 50 nm steps of the piezo stage. This was achieved by dispersing a low melting point agarose (TopVision, R0801) in warm (> 65 °C) distilled water and then adding a very dilute suspension of beads into the melt, before pipetting a small amount of sample onto a microscope slide and letting it set at room temperature. The sample was then mounted on the microscope, a single bead brought into focus, and measurements performed by moving the sample through a known distance via the piezo z-stage. Several different batches of beads were used with a range of different diameters; d = 2.1 µm (Polysciences Inc, 19508), d = 3 µm (Polysciences Inc, 17134), d = 4.5 µm (Polysciences Inc, 17135), nominal d = 5 µm (ThermoScientific 35–2, these beads were actually highly polydisperse, d = 5–8 µm ) and d = 9 µm (Invitrogen, N37464).

## Results and discussion

### Z-localisation

In order to perform microrheology measurements, an accurate localisation of the trapped bead is needed. In x and y directions, this is typically achieved by thresholding the image and then taking the centre of mass of the bead intensity profile in each dimension, as defined by Eq. (1). Here I_xy_ is the intensity of the pixel with indices x and y in each dimension and the sum is over all pixel indices.

1 $$\left( {x_{{Centre{ }of{ }Mass}} ,y_{{Centre{ }of{ }Mass}} } \right) = \left( {{ }\frac{{\Sigma_{x = 1}^{N} \Sigma_{y = 1}^{N} I_{xy} x}}{{\Sigma_{x = 1}^{N} \Sigma_{y = 1}^{N} I_{xy} }},{ }\frac{{\Sigma_{x = 1}^{N} \Sigma_{y = 1}^{N} I_{xy} y}}{{\Sigma_{x = 1}^{N} \Sigma_{y = 1}^{N} I_{xy} }}} \right)$$

**Figure 3:**
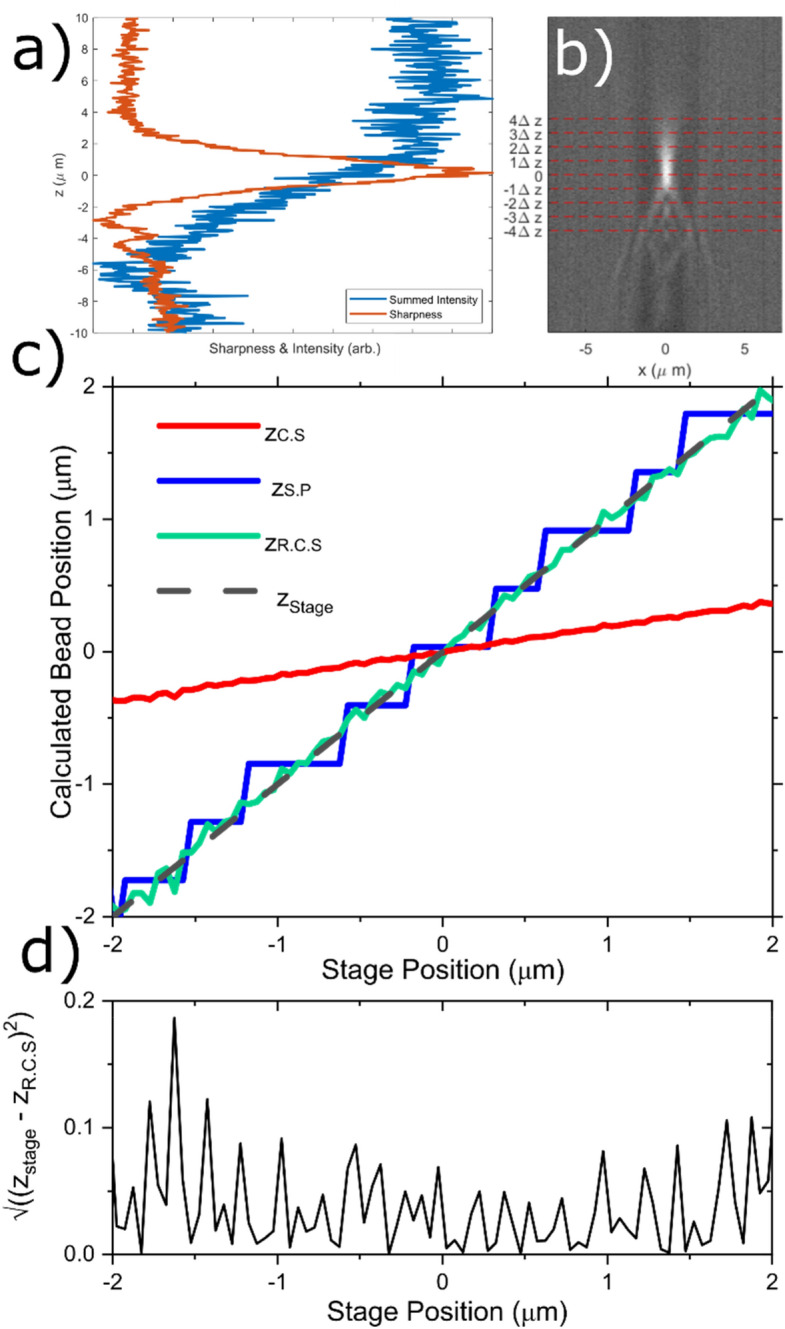
(**a**) Sharpness (orange) and summed intensity (blue) lines taken from the central plane images of a 3 µm diameter bead as it was moved through z, with steps of 50 nm. (**b**) Cross-sections taken through the centre of the image of the 3 µm bead in the central plane as the bead is moved through z. The red dashed lines correspond to the spacing of the different segmented planes. Contrast and brightness adjusted for clarity. (Matlab 2019b; MathWorks, Natick, MA, https://uk.mathworks.com/products/matlab.html) (**c**)The calculated z-position of the bead using the sharpest plane (z._S.P_, blue), centre of sharpness (z_C.S_, red), rescaled centre of sharpness (z_R.C.S_, green) approaches to determine the z position versus stage position in z. Stage position is also plotted as a dashed black line as a comparison. (**d**) Residuals of the z_R.C.S_ data shown in (**c**).

However, as shown in Fig. 3a, for a colloidal bead imaged in transmission, the sum of the image intensity has poor signal to noise ratio (blue curve) and does not peak when the bead is sharply in focus. Therefore, the centre of mass method used in x and y cannot simply be extended to the z-dimension, and a different approach must be used, as described below.

The image sharpness is defined by Eq. (2), which is a single metric that peaks when there are minimal aberrations and falls off rapidly as aberrations increase^38^. Our system has narrow spectral filtering, uses high quality optics and is carefully aligned to minimise aberrations, so the shape of the sharpness curve will be dominated by defocus. In Fig. 3a we show the sharpness curve for a bead being scanned in z (orange curve).

2 $$Sharpness(z)=\frac{{\Sigma }_{x=1}^{N}{\Sigma }_{y=1}^{N}\left({I}_{xyz}^{2}-{I}_{xyz}\right)}{{\left({\Sigma }_{x=1}^{N}{\Sigma }_{y=1}^{N}{I}_{xyz}\right)}^{2}}$$

In a previous work by this group^34^, a fixed bead was repeatedly moved by a known distance in z and the sharpness in each plane measured. This created a matrix of known sharpness values for each image plane in each stage position that was then used as a calibration dataset. By using statistical techniques based around maximum likelihood estimation, it was possible to calculate the position of this bead by comparing the measured Sharpness values to the calibration set and finding the z value most likely to produce those Sharpness values. This offered excellent precision, when used for sub-diffraction-limit sized beads rather than the 1 – 10 µm diameter beads commonly used in optical trapping based microrheology. Similar “look up table” based approaches have also been used for larger beads, and based on other metrics such as radial profile rather than sharpness^39–41^. However, regardless of the metric used, such approaches strongly rely on the ability of the user to generate their calibration by simply scanning their objective through z while holding the bead stationary. This is achievable for magnetic tweezers measurements, where the position of the trapped object can be controlled independently of the imaging plane, but for the majority of optical tweezers systems imaging and trapping are performed via the same objective lens which makes this far more challenging as it is not straight forward to move the bead relative to the image plane. A possible alternative would be to generate a calibration set using a different bead from the one which will be used for measurements. However, this depends on the assumption that the behaviour of the metric in the calibration set will be identical to those of the measurement. In reality, optical properties vary greatly depending on the depth being imaged at and the inhomogeneity of the sample microstructure (this is especially true of complex biological samples), and there will always be a degree of polydispersity in bead size (the Sharpness function is strongly dependent on bead size, as shown in Fig. S1).

Therefore, instead of using Sharpness directly as a metric for a look-up-table, we have explored the use of Sharpness as a weighting function for a centre of mass calculation in the z direction (similarly to how Intensity is used in the x–y plane) returning Eq. (3), where z_C.S_ is the centre of sharpness.

3 $${z}_{C.S}= \frac{{\Sigma }_{Plane=1}^{9} {z}_{Plane} Sharpness(Plane)}{{\Sigma }_{Plane=1}^{9} Sharpness(Plane)}$$

In Fig. 3c we show a comparison between z_C.S_ (red line) and the actual z position taken from the piezo controller (black dashed line), for a ~ 3 μm diameter bead fixed in agar and imaged in transmission with 15 ms exposure, as the stage is moved in the z direction in 50 nm increments. It is clear that z_C.S_ significantly underestimates the degree of motion of the bead, as the gradient of z_C.S_ (~ 0.2) is much lower than the gradient of 1 exhibited by z_stage_. This is because with only nine planes in z we are significantly undersampling the function (see Fig. 3b), and hence biasing z_C.S_ to be too close to zero.

An alternative approach is to identify the plane that has the highest Sharpness value at any given time (z_sharpest_) and take the z value of this object plane relative to the mid-plane to be the bead position. This method is accurate, but offers very low precision, equal only to the plane spacing. This can be improved by finding the plane with the second highest Sharpness value (z_2nd-sharpest_), and assuming that the bead must lay between these two planes, but closer to the sharpest plane. We define this as the ‘Sharpest Plane’ approach and z_S.P_ as in Eq. (4).

4 $${{z_{S.P} = z_{{Sharpest{ }}} - { }\left( {z_{{Sharpest{ }}} - z_{2nd - Sharpest} } \right)} \mathord{\left/ {\vphantom {{z_{S.P} = z_{{Sharpest{ }}} - { }\left( {z_{{Sharpest{ }}} - z_{2nd - Sharpest} } \right)} 4}} \right. \kern-\nulldelimiterspace} 4}$$

Equation (4) returns the same values as z_sharpest_ but shifted by ± Δz/4 depending upon whether z_2nd-Sharpest_ is the plane above or below z_sharpest_ This results in even step sizes of Δz/2 as the bead moves in z. Although z_S.P_ is more precise than z_sharpest_, it still only returns a resolution of half our plane spacing, as shown in Fig. 3b by the blue step-wise line. This approach lacks the spatial resolution of < 100 nm essential for microrheology studies.

**Figure 4:**
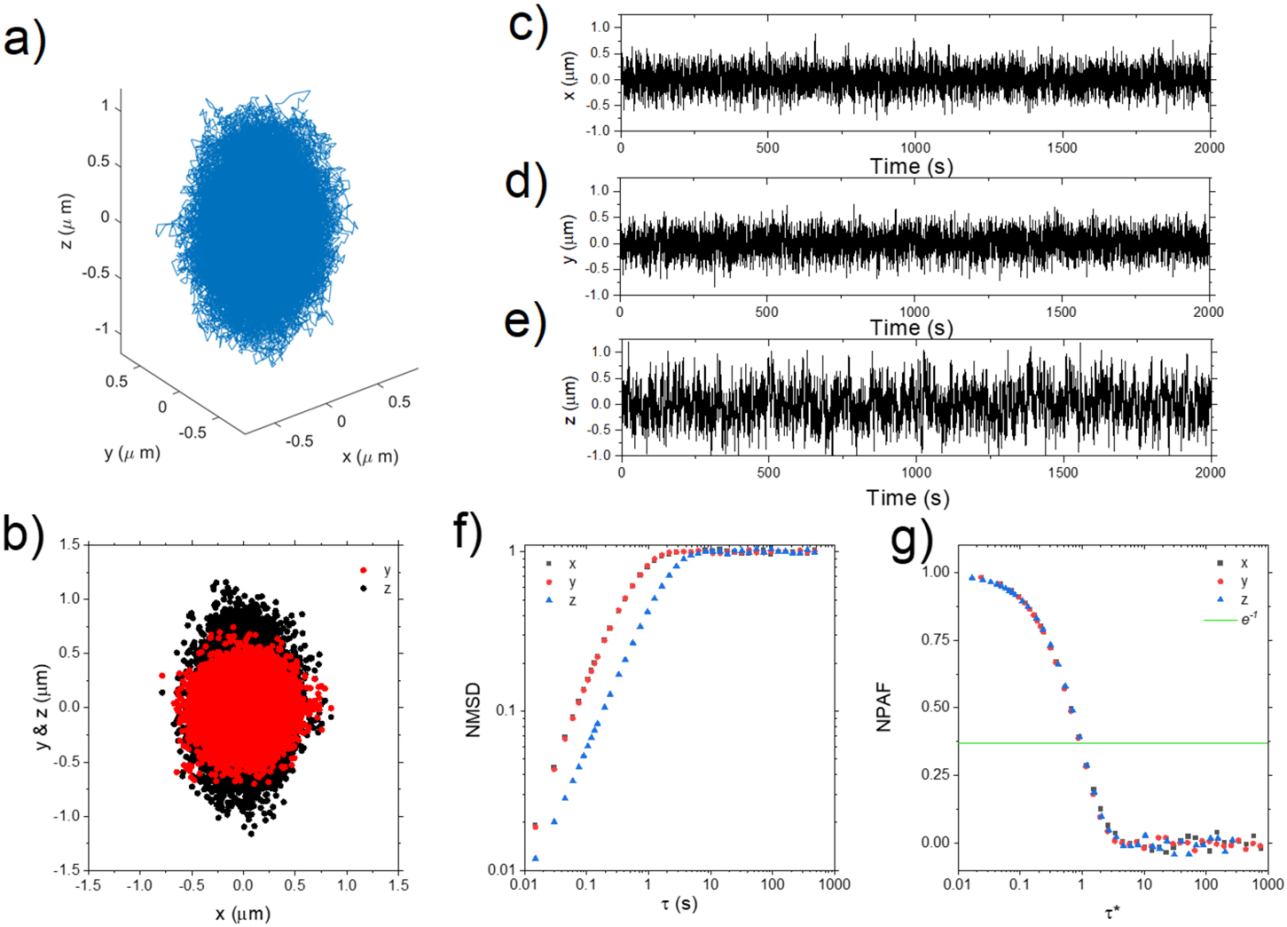
(**a**) 3D scatter plot of the trajectory of a ~ 7 µm diameter bead confined in space by an optical trap (Matlab 2019b; MathWorks, Natick, MA, https://uk.mathworks.com/products/matlab.html). (**b**) Projections of the trajectory on the x–y and x–z planes. The bead trajectory is drawn from the image analysis of ~ 100,000 frames. (**c**), (**d**) and (**e**) show the x, y, and z position respectively of the bead with time over the length of the experiment. (**f**) The bead NMSD versus lag-time τ evaluated for each dimension. (**g**) The particle NPAF for each dimension plotted against a dimensionless lag-time τ*^42^, derived from the scatter plots shown in (**a**). The solid line is at NPAF = e^-1^.

In order to achieve the required spatial resolution, we have combined the two methods outlined above. Specifically, we have used z_S.P_ as a calibration to linearly rescale z_C.S_. We have taken some additional steps to avoid the effects of non-linearity when the bead has moved far from equilibrium, and to reduce biasing (which we detail in Sect. 3 of the SI), to perform a linear fit of the stepped z_S.P_ data as a function of z_C.S_. For the data shown in Fig. 3 this returns a gradient of 5.26, which we take as a rescaling factor specific to a bead of this size under this illumination, i.e. z_R.C.S_ = 5.26 z_C.S_. It can be seen that z_R.C.S_ (green line) follows the actual stage position (black dashed line) very closely, as quantified by the residual plot. Notice that Fig. 3b was generated by moving the stage a total distance of only 4 µm. The calculated z position matches the stage position very well over this range, with a mean residual of ~ 40 nm. However, it must be highlighted that in actual hybrid optical trapping microrheology experiments where trap strengths are generally between 1 × 10^–6^ and 1 × 10^–8^ Nm^−1^, an optically trapped bead will typically move less than ± 1 µm in any dimension during a measurement performed at room temperature. This is confirmed in Fig. 4a,b, where the scatter plots of an optically trapped bead of ~ 4 µm radius are reported. Notice that the bead travels only ~ 1 µm in any direction from the centre of the optical trap. Therefore, when considering the accuracy of this method as it will be used in microrheology measurements, it is more relevant to consider the error in detecting the bead position for displacements of + /- 1 µm from the centre. In this region we obtain a mean absolute residual between z_R.C.S_ and z_stage_ of $\overline{{ \left| {z_{stage} {-} z_{R.C.S} } \right|}}$ ~ 30 nm for the d = 3 µm bead shown in Fig. 3d.

In order for this method to be effective, the plane separation must be large enough to adequately sample the peak and drop off of the sharpness function. This is demonstrated in Figs. S2–S5 where we show plots of the same type as Fig. 3c,d, but for beads of different sizes. For all the bead sizes explored, there is a region in which z_RCS_ scales linearly with stage position. For larger beads, within this central region the method is more accurate, and residuals are smaller. However, it must be noted that as bead size becomes larger, the range of z_stage_ values over which z_CS_ behaves linearly becomes narrower, even as the residuals within these regions decrease (see Fig. S6). This clearly demonstrates that one must optimize bead size for plane separation as larger beads will give less random noise but may introduce systematic error if a very weak optical trap is used, such that the bead regularly travels over a range wider than this linear region. For the Δz = 0.88 µm plane spacing employed in this work, we conclude that using a 9 µm bead may risk introducing systematic underestimation of z motion into MOT experiments, whereas the other beads tested would safely give linear behavior over the full extent of the optical trap. The ThermoScientific beads with diameters ranging from 5–8 µm offered a precision of $\overline{{ \left| {z_{stage} {-} z_{R.C.S} } \right|}}$ ~ 20 nm (see Fig. S4) within the ± 1 µm region, therefore, we have used beads from this batch for the measurements reported hereafter.

The proposed methodology depends on moving the bead up and down at the start of the measurement to generate the z_S.P_ function required to perform the in situ self-calibration procedure. This can be achieved in different ways depending on the nature of the sample under investigation. For instance, in the case of a bead embedded in a very stiff media capable of holding it in place (such as a gel), the use of any standard z-stage with step precision ~ Δz/2 or capable of continuous motion in z will allow this task to be accomplished. This is more complex for microrheology measurements of fluids, because the trapping laser and transmitted illumination light travel through the same objective. Therefore, moving the sample or the objective would not change the relative position of the trap (i.e., the bead) to that of the imaging planes. However, by moving lens L2 (as described in the method section and indicated in Fig. 1a) it is possible to adjust the position of the laser beam waist independent of the imaging plane. This allows us to move the bead over a distance of ± 4Δz, which is more than sufficient to obtain z_C.S_ values at a range of different values of z_S.P_, thus allowing a correct rescaling of z_C.S_ to generate z_R.C.S_. This is demonstrated in Fig. S6, which shows z_C.S_, z_S.P_ and z_R.C.S_ for the self-calibration step followed by the initial part of a microrheology measurement.

### 3D Microrheology with optical trapping

In order to corroborate our method, we performed preliminary microrheology measurements in water, a Newtonian fluid. Here we continue to operate at 67 Hz, which is adequate to analyse the viscous behavior of water. However, it important to highlight that, as there are no moving parts in the multiplane system, this speed is only limited by the camera acquisition rate. For instance, by using gratings with a larger etch period, or a shorter relay with gratings with higher curvature, we could reduce the field of view and thus the number of pixels in our images. We could then operate at ~ 500 Hz using the same camera. Figure 4a shows a 3D scatter plot of the trajectory of a bead confined within a 3D optical potential. The trajectory has been drawn by analysing 100,000 individual frames each recording 9 simultaneous z planes imaged during the measurement. This allows the visualization of the entire volume of the optical trap, which has a prolate spheroid shape stretched along the z-axis, due to the inherently weaker trap strength in the axial direction^43,44^. The spatial distribution along the three axial directions has a full-width-half-maximum of 0.45 µm, 0.45 µm and 0.76 µm in, x, y and z, respectively (Fig. 4b). The x, y and z coordinates versus time are plotted in Fig. 4c–e and do not show any significant drift, indicating a thermally and optically stable system. This is confirmed by looking at the Allan deviation of the bead position in each dimension which also shows no drift on the timescale of the experiment (see Fig. S7). Calculating the trap strength gives us $\kappa_{x} \cong \kappa_{y} \cong 1 \times 10^{ - 7} { }$ Nm^-1^ and $\kappa_{z} \cong 4 \times 10^{ - 8}$ Nm^-1^. The particle trajectory can be further exploited to evaluate its normalised mean square displacement (NMSD)^10^ as a function of elapsed time (τ) for each spatial dimension (x, y, z), as shown in Fig. 4f. We can see that the NMSD curves evaluated in the x and y directions overlay almost perfectly on top of each other, as expected for a spherical particle in an isotropic fluid and a well aligned optical system. The NMSD curve in the z direction, however, is offset in the time axis because of the weaker trap strength. This offset can be compensated for by following the analytical approach introduced by Tassieri et al.^42^, and plotting either the NMSD or the NPAF against a dimensionless lag-time, τ* = τκ/(6*πrη*_*s*_), which takes into account the bulk viscosity of the solvent (η_s_), as shown in Fig. 4g. We see that all the curves collapse onto a single master curve, as we are probing the same physical phenomenon, i.e. the Brownian motion of the same bead suspended in the same isotropic Newtonian fluid. Notably, this representation allows a direct measurement (‘*at a glance*’^42^) of the fluid relative viscosity ($\eta_{r} = \eta /\eta_{s}$), defined as the ratio of the fluid viscosity to that of the solvent. The relative viscosity will be the τ* value at which NPAF(τ*) = e^−1^; the latter is marked by the solid horizontal line in Fig. 4g. Notably, the abscissa of the intercept of this line with all three NPAFs occurs at τ* ≅ 1, meaning that the measured relative viscosity is 1 as expected for pure water. Measurements were repeated on 5 beads with d ~ 6–8 µm and obtained η^x^_r_ = 1.02 ± 0.04, η^y^_r_ = 1.01 ± 0.05, η^z^_r_ = 0.98 ± 0.07. This corroborates the overall effectiveness of the proposed experimental procedure.

## Conclusion

In this work we have introduced a novel 3D microrheology system named “OpTIMuM” that combines, for the first time, optical tweezers and multiplane microscopy. OpTIMuM allows us to perform particle tracking microrheology in three dimensions with ~ 20 nm accuracy along the laser beam axis, without the need to generate a ‘look up table’ and without knowledge of the bead size, the optical properties of the suspending medium, nor the use of any high precision positioner during the measurement. A straightforward manual adjustment to the position of a lens prior to measurement is all that is required to calibrate the system. Notably, since multiple planes are acquired simultaneously and not through a scanning process, the acquisition rate of our system is limited only by the camera frame rate. Using this system we were able to obtain the viscosity of water in all three spatial dimensions simultaneously. OpTiMuM provides a fast and effective method for exploring the full 3D micro-environment of an optically trapped bead. We envisage it could also be useful when measuring the forces involved in cell-surface interactions studies or when looking at force extension relationships of single molecules, and could open new routes to topographical, chemical and bio-mimetical surface engineering studies for biomedical applications.

## Supplementary Information

Supplementary Information.
