# Supplementary material for: Optical Tweezers with Integrated Multiplane Microscopy (OpTIMuM): a new tool for 3D microrheology
Source: Sci Rep. 2021 Mar 10;11:5614. doi: 10.1038/s41598-021-85013-y (PMC7946888; doi:10.1038/s41598-021-85013-y)
Supplement: Supplementary file 1 — Supplementary Information. [file 41598_2021_85013_MOESM1_ESM.docx]

Optical Tweezers with Integrated Multiplane Microscopy (OpTIMuM) – a new tool for 3D microrheology

Andrew B. Matheson ^1^, Lynn Paterson ^1^, Amanda J. Wright^2^, Tania Mendonca^2^, Manlio Tassieri ^3^, Paul. A. Dalgarno^1*^

^1^Institute of Biological Chemistry, Biophysics and Bioengineering, School of Engineering and Physical Sciences, Heriot Watt University, Edinburgh, EH14 4AS

^2^Optics and Photonics Research Group, Faculty of Engineering, University of Nottingham, Nottingham, NG7 2RD, UK

^3^Division of Biomedical Engineering, James Watt School of Engineering, University of Glasgow, 76 Oakfield Avenue, Glasgow, G12 8LT, UK

*p.a.dalgarno@hw.ac.uk

# Supplementary Information

## Sharpness for various bead sizes

Figure S1- Normalised Sharpness values for a range of bead sizes.

## z-localisation

Figure S2 – a) The calculated z-position of a 2.1 µm bead using the sharpest plane (z._S.P_, red) and rescaled centre of sharpness (z_R.C.S_, blue) approaches to determine the z position versus stage position in z. Stage position is also plotted as a dashed black line as a comparison. b) Residuals of the z_R.C.S_ data shown in a).

Figure S3 – a) The calculated z-position of a 4.5 µm bead using the sharpest plane (z._S.P_, red) and rescaled centre of sharpness (z_R.C.S_, blue) approaches to determine the z position versus stage position in z. Stage position is also plotted as a black line as a comparison. b) Residuals of the z_R.C.S_ data shown in a).

Figure S4 – a) The calculated z-position of a 5.8 µm bead using the sharpest plane (z._S.P_, red) and rescaled centre of sharpness (z_R.C.S_, blue) approaches to determine the z position versus stage position in z. Stage position is also plotted as a black line as a comparison. b) Residuals of the z_R.C.S_ data shown in a).

Figure S5 – a) The calculated z-position of a 9 µm bead using the sharpest plane (z._S.P_, red) and rescaled centre of sharpness (z_R.C.S_, blue) approaches to determine the z position versus stage position in z. Stage position is also plotted as a black line as a comparison. b) Residuals of the z_R.C.S_ data shown in a).

Figure S6 - Range over which z_cm_ behaves linearly and the mean residual within this region, versus bead diameter.

## Implementation of self-calibration step

Figure S7 - z position for the first 200 s of the measurement shown in Fig 4. The first ~ 80 s is the self-calibration step where the bead is moved up and down (by moving lens L2 shown in Figure 1a. On completion of the measurement the red z_C.S_ trace is rescaled to match the shape of the black z_S.P_ trace, creating the blue z_R.C.S_ that is an accurate measure of z-position.

As described in the main text, we have two metrics

$z_{S.P}=z_{Sharpest}- (z_{Sharpest}-z_{2nd-Sharpest})$/4

And

$z_{C.S}= \frac{\Sigma z_{plane} Sharpness(P)}{\Sigma Sharpness(P)}$

Rather than directly fit one of these functions against the other to get a scaling factor, we perform some intermediate steps to improve the accuracy of the fit.

Looking at Fig S7 we see that when |z_R.C.S_ | and |z_S.P_ |> 1, they begin to plateau, for instance in the time period 20 s < t < 60 s. This is due to the same non-linearity which we see at high and low values of Stage Position as shown in Fig S3-S5. To avoid being affected by this non-linearity we begin by selecting only the frames where -2Δz < z_S.P_< 2Δz for the rescaling to ensure we are in a region where z_C.S_ behaves linearly.

We then perform some further steps to reduce biasing due to there being more measurements taken at some values of z_S.P_ than at others. To do so we identify only the points at which z_S.P_ is changing as follows.

Firstly we take the derivative of z_S.P_ and identify all the frames at which $\frac{\partial z_{S.P}(t)}{\partial t}\neq0.$

Having identified these points which we call z_steps_ we then assume that at these points of transition the actual position lies half way between z_steps_ and the z_S.P_ immediately before the transition. This is equivalent to saying

$${z'(t)}_{steps}={z(t)}_{steps}-\frac{\Delta t}{2}\frac{\partial z_{S.P}(t)}{\partial t}$$

The reason to do this is to ensure that frames at which a bead has switched from z_S.P_ = 4 to z_S.P_ = 5 are assigned the same value of z’ as one where it goes from z_S.P_ = 5 to z_S.P_ = 4.

We then take the average value of z_C.S_ for each different value of z’_steps_, which we can do as z’_steps_ is a discrete rather than continuous variable.

We then perform a linear fit of z’_steps_ vs z_C.S_ such that

$$z_{steps}^{'}=A z_{C.S}+B$$

A is now our scaling factor that we can use to scale z_C.S_ to give

$$z_{R.C.S}=A z_{C.S}$$

Full code showing the implementation is available on request.

## Allan Deviation

Figure S8 - Allan deviation for the bead during microrheology measurements shown in Fig.4.
